# Palliative Osimertinib Rechallenge Achieving Rapid Improvement in Leptomeningeal Carcinomatosis After Prior Osimertinib‐Induced ILD

**DOI:** 10.1002/rcr2.70467

**Published:** 2026-01-12

**Authors:** Akina Nigi, Keisuke Iwamoto, Hidetoshi Itani, Shigeto Kondou

**Affiliations:** ^1^ Department of Respiratory Medicine Japanese Red Cross Ise Hospital Ise Mie Japan

**Keywords:** EGFR mutation, interstitial lung disease, leptomeningeal carcinomatosis, osimertinib, rechallenge

## Abstract

A patient with EGFR‐mutant lung cancer developed leptomeningeal carcinomatosis years after osimertinib‐induced ILD. With no other treatment options, palliative osimertinib rechallenge led to rapid neurological improvement and radiological response, without ILD recurrence. This case highlights the potential of carefully monitored osimertinib rechallenge for symptomatic relief in exceptional cases.

## Graphical abstract

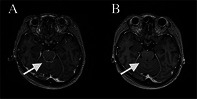

A 60‐year‐old woman with stage IVB EGFR‐mutated lung adenocarcinoma and prior brain metastases was initially treated with osimertinib but discontinued therapy after developing CTCAE grade 2 interstitial lung disease (ILD). She subsequently received carboplatin, pemetrexed and bevacizumab, followed by maintenance erlotinib without further ILD. Four and a half years later, she developed multiple brain metastases and cauda equina metastasis, which were treated with radiotherapy; however, neurological symptoms progressed, and MRI later demonstrated advancement to leptomeningeal carcinomatosis (LMC) (Figure 1A). Despite four cycles of docetaxel plus ramucirumab, her dysphagia, dysarthria and gait disturbance worsened.

**FIGURE 1:**
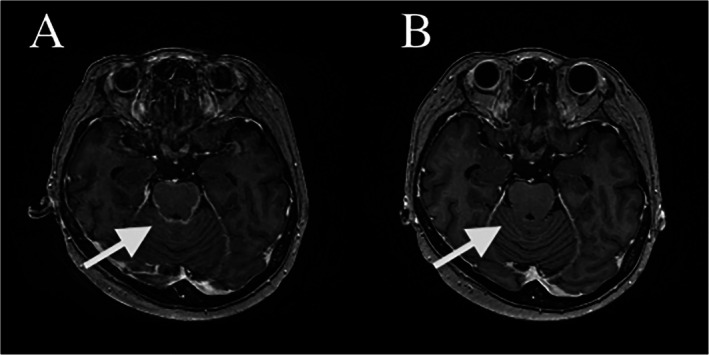
(A) Baseline contrast‐enhanced MRI showing diffuse leptomeningeal carcinomatosis (arrow). (B) Fourteen days after palliative osimertinib rechallenge, leptomeningeal enhancement markedly improved (arrow).

Although rechallenge with the same class of EGFR‐TKI is generally avoided after ILD, the patient elected palliative re‐introduction of osimertinib. Neurological improvement was observed within 1 week. Follow‐up MRI 14 days after rechallenge showed marked radiologic improvement of LMC (Figure 1B), while parenchymal brain metastases remained unchanged.

Erlotinib efficacy had diminished before progression, yet the superior cerebrospinal fluid penetrance of osimertinib compared with erlotinib may explain the observed clinical response [1]. No ILD recurrence was noted 2 months after restarting therapy.

This case suggests that, in selected patients with rapidly progressive LMC and limited options, carefully considered palliative osimertinib rechallenge may provide meaningful symptomatic and radiologic benefit despite prior EGFR‐TKI–induced ILD.

## Funding

The authors have nothing to report.

## Consent

The authors declare that written informed consent was obtained for the publication of this manuscript and accompanying images using the consent form provided by the Journal.

## Conflicts of Interest

The authors declare no conflicts of interest.

## Data Availability

No additional data are available beyond what is included in the article.
